# Shp1 in Solid Cancers and Their Therapy

**DOI:** 10.3389/fonc.2020.00935

**Published:** 2020-06-11

**Authors:** Alessia Varone, Daniela Spano, Daniela Corda

**Affiliations:** ^1^Institute of Biochemistry and Cell Biology, National Research Council, Naples, Italy; ^2^Department of Biomedical Sciences, National Research Council, Rome, Italy

**Keywords:** Shp1, cancer, signaling, tyrosine phosphatase, cancer therapy, TCGA

## Abstract

Shp1 is a cytosolic tyrosine phosphatase that regulates a broad range of cellular functions and targets, modulating the flow of information from the cell membrane to the nucleus. While initially studied in the hematopoietic system, research conducted over the past years has expanded our understanding of the biological role of Shp1 to other tissues, proposing it as a novel tumor suppressor gene functionally involved in different hallmarks of cancer. The main mechanism by which Shp1 curbs cancer development and progression is the ability to attenuate and/or terminate signaling pathways controlling cell proliferation, survival, migration, and invasion. Thus, alterations in Shp1 function or expression can contribute to several human diseases, particularly cancer. In cancer cells, Shp1 activity can indeed be affected by mutations or epigenetic silencing that cause failure of Shp1-mediated homeostatic maintenance. This review will discuss the current knowledge of the cellular functions controlled by Shp1 in non-hematopoietic tissues and solid tumors, the mechanisms that regulate Shp1 expression, the role of its mutation/expression status in cancer and its value as potential target for cancer treatment. In addition, we report information gathered from the public available data from The Cancer Genome Atlas (TCGA) database on Shp1 genomic alterations and correlation with survival in solid cancers patients.

## What is Shp1?

Src homology region 2 (SH-2) domain-containing phosphatase 1 (Shp1) is a non-receptor tyrosine phosphatase encoded by the *PTPN6* gene that is located on human chromosome 12p13 and contains two promoter regions (within exon 1 and 2), giving rise to two forms of Shp1 which differ in their N-terminal amino acid sequences but have a similar phosphatase activity. Promoter I is active in non-hematopoietic cells, while promoter II in hematopoietic-derived cells (1, 2); in some epithelial cancer cells both promoters may function and generate various Shp1-alternative transcripts (3). The two Shp1 isoforms show different subcellular localizations: form I is mainly located in the nucleus, while form II is in the cytoplasm (2), suggesting that they have different targets.

Shp1 is a 595 amino acid protein composed of two tandem N-terminal SH2 domains (N-SH2 and C-SH2), a classic catalytic protein tyrosine phosphatase (PTP) domain, and a C-terminal tail containing several phosphorylation sites (4–6). Its crystal was first resolved by Yang et al. (4) and revealed a structure in which the N-SH2 is bound to the catalytic site of the protein through charge-charge interaction (4). In this auto-inhibited inactive state the access of substrates to the active site is prevented, but binding of phosphotyrosine residues to the SH2 domains causes a conformational change that impairs the interaction between the N-SH2 and the catalytic domains. This opens the conformation to allow the access of substrate and is further stabilized by new interactions between SH2 domains and the catalytic domain (6). These molecular rearrangements determine a sophisticated regulatory mechanism controlled by substrate recruitment.

An additional mechanism of activation is mediated by the phosphorylation of amino acids within the C-terminal tail. So far, three phosphorylation sites have been found, two tyrosine (Tyr536 and Tyr564) and a serine (Ser591) residues. Tyr536 and Tyr564 become phosphorylated upon various stimuli (i.e., insulin stimulation or apoptosis inducers), giving rise to an increased Shp1 activity (7–9). The molecular mechanism is not clear, although it has been proposed that Tyr phosphorylations could lead to interaction with the N-SH2 domain, releasing the inhibitory effect of this domain on the PTPase activity (10). Shp1 activity can also be negatively regulated by protein kinase C (PKC) or mitogen-activated protein kinases (MAPKs) through phosphorylation at Ser591, whose mechanism of inhibition has not been well-characterized (11).

## Shp1 and Cancer

Protein-tyrosine phosphorylation is a reversible post-translational modification, tightly regulated by both kinases and phosphatases. Any deviation in the phosphorylation/dephosphorylation balance can promote the intracellular accumulation of tyrosine-phosphorylated proteins, which cause an altered regulation of cellular processes including cell growth, migration, invasion, differentiation, survival, and cellular trafficking (12, 13). In this scenario, Shp1 acts as a classical tumor suppressor, mainly involved in the homeostatic maintenance of potentially all these processes. Shp1 function is indeed altered in both solid and hematological human cancers through somatic mutations or epigenetic mechanisms. Besides its well-documented role in the regulation of hematopoietic cell biology [widely discussed in recent reviews to which the reader is referred (14–16)], Shp1 has now been correlated to a number of signal transduction pathways relevant to cancer pathogenesis and progression.

**Figure 1:**
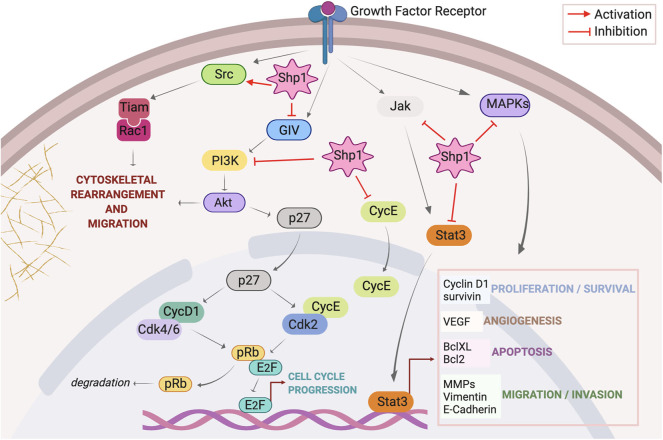
Overview of signaling pathways regulated by Shp1. Activation of growth factor receptors initiates signaling through Src, PI3K/Akt, JAK/STAT, and MAPKs axes. Once activated Src triggers a signal pathway that culminates with the assembly of Tiam/Rac1 complex at the plasma membrane and consequent formation of peripheral ruffles with stimulation of cell motility; Shp1 dephosphorylates Src on the inhibitory pTyr527 thus activating this cascade. Membrane receptor activation triggers the tyrosine phosphorylation of GIV that directly binds and activates PI3K; this in turn activates Akt inducing cytoskeletal rearrangements. Shp1 dephosphorylates GIV and inhibits Akt activation via the GIV-PI3K axis. Akt also phosphorylates p27(kip1) (p27) at Thr157, promoting its cytoplasmic localization and abolishing its inhibitory effect on cell cycle components like cyclin D1-Cdk 4/6 and cyclin E-Cdk 2 complexes; this results in increased pRb phosphorylation and dissociation from E2F, stimulation of transcriptional activity with consequent cell cycle progression. Shp1 blocks p27(kip1) nuclear localization through the regulation of PI3K/Akt activity. Shp1 also controls cell cycle progression regulating cyclin E localization. Following receptor stimulation, activated JAK phosphorylates STAT3, resulting in the translocation of activated STAT3 (p-STAT3) to the nucleus; Shp1 can directly dephosphorylate STAT3 or its upstream JAK thereby hampering STAT3-regulated cellular proliferation and survival, angiogenesis, apoptosis, migration, and invasion. Finally, Shp1 is involved in the attenuation of growth factors induced cell proliferation trough inhibition of MAPKs pathway.

Here we discuss the recent knowledge on Shp1 pathways relevant to cancer (Figure 1), its alteration in tumors and relationship with the clinic including some therapeutic approaches and known drug candidates that target this protein.

## Shp1 in Cell-Cycle Progression

Shp1 is generally considered as a negative regulator of cell proliferation, but current knowledge provides a more complex view of its role in cell signaling pathways, so that this enzyme can function both as a positive or negative mediator, depending on the target molecules and the specific cell type. Evidence suggests a role for Shp1 in the regulation of cell cycle, due to the control of mitogenic pathways activated by receptor tyrosine kinases or to its ability to modulate cell cycle components through direct interaction (17).

In endothelial cells Shp1 activation by tumor necrosis factor alpha (TNFα) inhibits proliferative responses to growth factors such as vascular endothelial growth factor (VEGF) and epidermal growth factor (EGF), through attenuation of extracellular signal-regulated kinase (ERK) phosphorylation and MAPKs pathway. Here the overexpression of dominant-negative Shp1 prevents TNFα-induced inhibition of cell growth (18). In hepatic stellate cells Shp1 mediates the anti-proliferative signals downstream of platelet-derived growth factor (PDGF) receptor-β by regulating both proliferative and survival markers, such as the expression of cyclin D1 and the phosphorylation of Akt and ERK1/2 kinases (19). Importantly, this effect can be positively regulated by molecules that stimulate Shp1 expression, suggesting a potential therapeutic implication of Shp1 in liver fibrosis treatment (19). Along these lines, other studies report the involvement of Shp1 in the mechanisms of action of anti-proliferative stimuli, such as somatostatin. Somatostatin negatively regulates insulin signaling by controlling Shp1 recruitment to the insulin receptor. As for the PDGF receptor, the recruitment of Shp1 to the membrane induces receptor dephosphorylation and inactivation leading to the termination of insulin signaling and relative mitogenic response (20). Besides this mechanism, Shp1 can further regulate somatostatin signaling through the up-regulation of the cyclin-dependent kinase (Cdk) inhibitor p27(Kip1), inhibition of Cdk4 and Cdk2 activities with a consequent dephosphorylation of the Retinoblastoma protein and induction of cell-cycle arrest (21).

A mechanism alternative to the receptor regulation through which Shp1 controls cell cycle is the direct interaction with cell-cycle components. In intestinal epithelial cells, Shp1 expression and enzymatic activity induce cell cycle block and differentiation (22). Indeed, the overexpression of Shp1 in intestinal crypt cells decreases the expression of cyclin D1 and c-myc genes through the inhibition of E2F-dependent transcriptional activity, thus affecting the transition from G0/G1 to S phase (22). Later on, the same authors identified Cdk2 as a novel Shp1 interactor in epithelial cells (23). In proliferating intestinal cells Cdk2 interacts with Shp1 and promotes its proteasome-dependent truncation through direct Cdk2-dependent phosphorylation, similarly to the Cdk2-mediated degradation of other cell cycle regulators, such as p27(Kip1) (24) and cyclin E (25). Other groups also confirmed Cdk2-Shp1 interaction in different cell systems (26, 27). In a reciprocal manner Shp1 regulates Cdk2 localization and activity modulating cyclin E levels (28). This Cdk2 regulation has been proposed as a mechanism through which Shp1 regulates p27(Kip1), since Cdk2 phosphorylates p27(kip1) and promotes its degradation (28).

However, the involvement of p27(kip1) in the Shp1-mediated regulation of cell proliferation is controversial. In CHO cells, Shp1 mediates the anti-proliferative effect of somatostatin by the upregulation of p27(kip1) (21). Similarly, in microvascular endothelial cells Shp1 mediates the anti-proliferative activity of the tissue inhibitor metalloproteinase-2 (TIMP2) by increasing the *de novo* synthesis of p27(kip1) with the parallel inhibition of Cdk2 and Cdk4 activities (29).

Several studies also describe positive effects of Shp1 on mitogenic signaling. In HEK293 cells the overexpression of a catalytically-inactive mutant of Shp1 reduces cell growth and DNA synthesis through the suppression of mitogen-activated pathways (30). The same in ovarian cancer cells where the inhibition of Shp1 activity reduces tumor growth by increasing intracellular levels of the Cdk2/p27(kip1) and Cdk2/Shp1 complexes (26).

Therefore, the role of Shp1 in the regulation of cell cycle is still quite debated and further studies are needed to clarify its contribution to the control of cancer cell cycle. However, these apparent contradictions in Shp1 function might be due to the overexpression of dominant-negative mutants which could have non-specific effects. Nevertheless, we cannot rule out that the different Shp1 activity may lie in a differential interaction with tissue-specific proteins.

## Shp1 in Cell Motility and Invasion

Shp1 has been recently correlated to a number of processes controlling epithelial to mesenchymal transition (EMT), cell migration and invasion. Studies conducted in several cancers define Shp1 as a negative regulator of these processes, particularly controlling the signal transducer and activator of transcription (STAT) pathway (31). Hyperactivation of oncogenic STAT3 has been observed in various malignant tumors where it controls cellular signaling regulating proliferation (e.g., cyclin D1), cell survival (e.g., Bcl-xl, Bcl2, survivin, Mcl-1 and c-Myc), but also migration and invasion [e.g., Rho, Rac, matrix metalloproteinase (MMP)-2, MMP-7, and MMP-9], as well as angiogenesis [e.g., VEGF; (32, 33)]. The major phosphorylation sites in STAT3 are Tyr705 and Ser727; Shp1 has been shown to directly dephosphorylate STAT3 on Tyr705 thus silencing the downstream pathway (34).

Protein levels of Shp1 indeed negatively correlate with p-STAT3Tyr705 and EMT markers in several hepatocellular carcinoma (HCC) cell lines (35). Cells not expressing Shp1 show higher levels of p-STAT3Tyr705 and vimentin, but a significantly lower or also loss of E-cadherin expression. As a result, these cells have a greater migratory and invasive capacity compared to other cell lines. Moreover, the Shp1 re-overexpression abolishes transforming growth factor (TGF)-β1-induced STAT3Tyr705 phosphorylation, EMT and invasion. The relationship between Shp1 and EMT was further confirmed in colorectal cancer (CRC) cells where Shp1 controls TGF-β1-induced EMT by suppressing p-STAT3Tyr705 (36). Several authors also reported how Shp1 negatively regulates the Janus kinase (JAK)-STAT pathway targeting p-STAT3Tyr705 in different systems, downregulating its target genes including those involved in invasion and metastasis (37–43).

Other studies demonstrate that Shp1 regulates migration and invasion through mechanisms other than the STAT3 pathway. Gα-interacting vesicle-associated protein (GIV) is a multidomain protein required by growth factors to enhance Akt activation and directly link Akt to the actin cytoskeleton, actin remodeling and cell migration (44). Shp1 antagonizes phospho-GIV by directly binding and dephosphorylating two phosphotyrosine residues within the C-terminal tail of GIV that serves as docking sites for p85α-regulatory subunit of phosphoinositide 3-kinase (PI3K). With this mechanism Shp1 may abrogate phospho-GIV-dependent enhancement of PI3K-Akt activity, resulting in the suppression of TGF-β1-induced EMT (44).

Our group has recently shown that Shp1 regulates actin remodeling and cell motility through yet another mechanism involving Src kinase (45). We identified Shp1 as the cellular receptor of the bioactive metabolite glycerophosphoinositol-4 phosphate (GroPIns4*P*) and as a novel component in the signaling pathway activated by EGF to control cell motility (45). In NIH3T3 cells, activation of the EGF receptor leads to activation of the cytosolic phospholipase A_2_ and results in the hydrolysis of phosphatidylinositol-4 phosphate and formation of GroPIns4*P* that binds to Shp1 and promotes its association with Src. This in turn activates Src by direct dephosphorylation of the inhibitory phosphotyrosine in position 530 and triggers a signaling cascade which ends with the formation of the Tiam/Rac complex at the cell membrane and the concomitant induction of plasma membrane ruffles leading to cell motility (45, 46).

## Shp1 in Cell Death and Apoptosis

Along with the cellular processes described above, Shp1 regulates cell death and apoptosis. The antitumor-action of Shp1 is mainly due to its negative regulation of STAT3 oncogenic signaling, through direct regulation of JAK (47) and STAT3 (48). Indeed, besides being a master regulator of a plethora of cellular functions, STAT3 controls a series of target genes encoding for anti-apoptotic and proliferation-associated proteins (such as Bcl-xL, Bcl-2, cyclin D1, and Survivin) (33).

Numerous studies report how Shp1-mediated STAT3-downregulation represents a promising anti-cancer strategy to inhibit tumor growth and induce apoptosis in cancer cells. Indeed, a number of chemotherapeutic drugs (49–52) as well as Shp1-targeting natural compounds (40, 53, 54) induce Shp1-mediated dephosphorylation of p-STAT3Tyr705; this downregulates STAT3 transcriptional activity causing a block of tumor cell proliferation and induction of apoptosis. These compounds might have therapeutic relevance in cancer therapy through Shp1 activation and/or stabilization as discussed in the next sections.

## Shp1 Alteration in Human Solid Cancers

Genomic alterations as well as epigenetic changes are critical for cancer onset, development and progression (55–58). To shed light on the Shp1 contribution to tumorigenesis we mined The Cancer Genome Atlas (TCGA) data for mutations and alterations of *PTPN6* gene. *PTPN6* genetic alterations, including mutations, fusion, amplification, deep deletion and multiple alterations, are found in several types of cancer. The total percentage of *PTPN6* alterations in cancer amounts at 2.7% (with amplifications and mutations as the most frequent ones) whereas uterine carcinosarcoma (7.01%), testicular germ cell cancer (6.04%), ovarian cancer (5.82%), and melanoma (5.18%) show the highest frequency of *PTPN6* genomic alterations.

As reported in Table 1, tumor-associated mutations may occur in the whole Shp1 sequence with most mutations (42.27%) in the phosphatase domain; this implies that each specific protein region may be pathologically relevant to cancer development. Most of these mutations (81.4%) are reported as missense, but to our knowledge there are no data regarding their effects on Shp1 function. The TCGA database reports just a few non-sense mutations that generate non-functional Shp1-truncated products. Finally, the major genomic alteration described in the *PTPN6* gene is the loss of heterozygosity, mainly observed in HCC patients (59).

**Table 1:** PTPN6 gene mutations.

**N-SH2 domain**	**Missense mutations**	**Allele frequency**	**Tumor**
	**R30Q**	**0.39**	**Mucinous adenocarcinoma of the colon and rectum**
	**R44K**	**0.47**	**Cutaneous melanoma**
	**D59N**	**0.44**	**Diffuse large B-cell lymphoma, NOS**
	**K68T**	**0.47**	**Colon adenocarcinoma**
**Linker region between N- and C-SH2 domains**	**Missense mutations**	**Allele frequency**	**Tumor**
	D90N	0.42	Uterine endometrioid carcinoma
	**Nonsense mutations**	**Allele frequency**	**Tumor**
	Q83^*^	0.33	Diffuse large B-cell lymphoma, NOS
**C-SH2 domain**	**Missense mutations**	**Allele frequency**	**Tumor**
	A120T	0.3	Stomach adenocarcinoma
	**In frame insertion**	**Allele frequency**	**Tumor**
	V169_M170dup	0.36	Uterine carcinosarcoma/Uterine Malignant mixed mullerian tumor
**Linker region between C-SH2 and PTP domains**	**Missense mutations**	**Allele frequency**^†^	**Tumor**
	E201K	0.43	Head and neck squamous cell carcinoma
	T216M; A220V	0.35; 0.31	Uterine endometrioid carcinoma
	E237K	0.39	Cutaneous melanoma
	F248S	0.48	Mucinous stomach adenocarcinoma
	Q266L	0.42	Hepatocellular carcinoma
**PTP domain**	**Missense mutations**	**Allele frequency**^†^	**Tumor**
	A323T	0.39	Mucinous stomach Adenocarcinoma
	V362I; L405M; R407W; Y412C	0.81; 0.35; 0.44; 0.43	Uterine endometrioid carcinoma
	P376L; G477S	0.59; 0.34	Cutaneous melanoma
	E389K; L478M	0.31; 0.32	Colon adenocarcinoma
	D419G	0.49	Diffuse large B-cell lymphoma, NOS
	Q438K	0.41	Glioblastoma multiforme
	**Nonsense mutations**	**Allele frequency**	**Tumor**
	G291^*^	0.38	Lung squamous cell carcinoma
	E384^*^	0.32	Uterine endometrioid carcinoma
	**Splice**	**Allele frequency**	**Tumor**
	X403_splice	0.31	Cutaneous melanoma
**C-terminal tail**	**Missense mutations**	**Allele frequency**	**Tumor**
	E517G	0.45	Uterine endometrioid carcinoma
	R554C	0.56	Colon adenocarcinoma
	**Splice**	**Allele frequency**	**Tumor**
	S528=	0.36	Colon adenocarcinoma
	X558_splice	0.47	Uterine endometrioid carcinoma

† *The mutation and the corresponding allele frequency are listed in the same order*.

* *The sense codon is mutated in a stop codon. Mutations listed in this table have an allele frequency of ≥0.3%*.

Epigenetic silencing of Shp1 expression is also observed in several cancer types, often due to the presence of hypermethylated CpG islands in the *PTPN6* promoter. Although this alteration regards mainly hematological malignancies (60–64), it has been shown to occur also in a set of solid tumors, including esophageal squamous cell carcinoma (65), gastric adenocarcinoma (66), HCC (59), breast (67), and endometrial carcinoma (68). In hematopoietic cancers, besides the hypermethylation, enrichment of histone markers of silencing (e.g., trimethylation at lysine 9 of histone H3, trimethylation at lysine 27 of histone H3 and acetylation at lysine 9 of histone H3) is detected at the *PTPN6* promoter (69), thus implying that also these histone modifications intervene in *PTPN6* expression regulation. Although a similar mechanism has not yet been reported for solid tumors, we cannot exclude that it contributes, jointly to the hypermethylation, to the *PTPN6* gene silencing.

Taken together, these alterations result in a diminished or abolished expression of Shp1 in most cancer cell lines and tissues examined; this deregulates the oncogenic pathways described above promoting malignant transformation.

## Shp1 Expression and Clinical Correlation in Solid Cancers

According to our analysis of the TCGA database, cancer patients with *PTPN6* genetic alteration(s) have a worse overall survival compared to those without *PTPN6* alteration(s). To our knowledge, no data are available regarding the correlation between *PTPN6* genetic alteration(s) and the progression-free survival in cancer patients. We therefore analyzed the TCGA data and found that the relationship between Shp1 expression and overall survival may be different in different solid cancers. Thus, higher Shp1 mRNA levels are associated to a worse overall survival outcome in kidney-renal clear-cell carcinoma and rectum adenocarcinoma patients, and to a better survival outcome in bladder carcinoma, breast cancer, cervical squamous cell carcinoma, kidney renal papillary cell carcinoma, lung adenocarcinoma, pancreatic ductal adenocarcinoma, sarcoma, stomach adenocarcinoma, and uterine corpus endometrial carcinoma patients. No correlation between Shp1 expression and overall survival was detected in other cancer types. Therefore, although *PTPN6* is generally considered as a tumor suppressor gene, Shp1 could have different roles in tumorigenesis depending on the different biological background. This could explain the opposite correlations between Shp1 expression and overall survival observed in different type of cancers.

As mentioned, Shp1 expression changes in different types of cancer. In the hematological malignancies and in some solid tumors Shp1 expression is reduced or absent as a result of *PTPN6* promoter hypermethylation (59–68). On the contrary, increased Shp1 levels are detected in a subset of high-grade breast tumors (70) and in ovarian cancers (71).

Several studies have been devoted to assess the prognostic and diagnostic value of Shp1 expression and promoter methylation, with the aim of identifying new biomarkers of cancer development. Shp1 decreased expression and *PTPN6* hypermethylation are associated with tumor staging, pathological differentiation and poor survival in patients with esophageal squamous cell carcinoma (65). In endometrial carcinoma, *PTPN6* hypermethylation is associated with age and tumor differentiation, while no correlation is found with muscular infiltration depth and lymphatic metastasis (68). In prostate cancer and high-grade glioma, *PTPN6* promoter methylation and reduced expression of Shp1 correlate with increased malignancy and poor prognosis (72–75). Moreover, the *PTPN6* high-methylation level is associated with early relapse of non-small cell lung cancer (NSCLC) (76). In addition, the *PTPN6* high-methylation level of lymph nodes from CRC patients (77) and from stage I NSCLC patients (76) is associated with recurrence and/or poor prognosis.

Interestingly, a good correlation of *PTPN6* methylation between cell-free circulating DNA from plasmas/sera and matched tumoral tissue is observed in glioma patients (75). Similarly, *PTPN6* promoter methylation is significantly increased in plasma from NSCLC patients compared to healthy controls (78); its methylation level is also associated with the rate of survival in advanced NSCLC (78). These data suggest that *PTPN6* methylation in plasma, in combination with clinical analysis, may be a promising biomarker for NSCLC diagnosis and prognosis.

## Therapeutic Implications and Emergent Drugs

From all the above it appears that Shp1 represents an attractive target for drug development in cancer treatment. Inhibitors targeting the Shp1 phosphatase activity have been under development for some times, and some have now entered preclinical studies, including NSC-87877, sodium stibogluconate (SSG), tyrosine phosphatase inhibitor 1 (TPI-1), and suramine; however, only a few of them have been shown to be active in experimental tumor models (79). SSG has been through Phase I trials for both malignant melanoma (NCT00498979) and advanced malignancies (NCT00629200); the drug was administrated in combination with interferons followed or not by chemotherapy treatment. Unfortunately, no effect was seen against tumor development (80, 81), with the most common toxic side-effects being thrombocytopenia, elevated serum lipase, fatigue, fever, chills, anemia, hypokalemia, pancreatitis, and skin rash (observed in up to 68% of patients). At present, no Shp1 inhibitor is under Phase II trial.

Shp1 might represent an interesting target to modulate oncogenic STAT3 activities and thus inhibit tumor growth, promote apoptosis and prevent chemo- and radio-resistance even in tumors in which it is not mutated (82, 83). Guggulsterone [a phytosteroid extracted from the guggul plant (84)], plumbagin [a vitamin K3 analog derived from a medicinal plant (38, 85)], and morin [a flavonol extracted from mulberry figs and old fustic (86)] modulate STAT3 activities through induction of Shp1 expression; instead, the multi-targeted kinase inhibitors dovitinib (87), sorafenib (50), and its derivatives (88, 89) such as regorafenib (90, 91) and SC-60 (92) have antitumor activities by direct binding to, and enhancement of, Shp1 phosphatase activity.

Despite the pharmacological potential reported for these molecules in *in-vitro* and *in-vivo* studies performed on multiple cancer types (including leukemia, head and neck cancer, melanoma, and HCC), only sorafenib and regorafenib have been so far approved by the Food and Drug Administration for cancer treatment. Shp1 inhibitors instead need to be further developed to exploit the inhibition of the Shp1-affected pathways within cancer cells and/or enhance the efficacy of existing chemotherapeutic agents.

## Shp1 and Cancer Immunotherapy

The repertoire of Shp1 functions is continuously expanding to include the modulation of the tumor microenvironment, thus suggesting new potential therapeutic implications. In recent years cancer immunotherapy, which exploits T-cells to arm the immune system against tumoral cells, has shown promising results based on a multitude of targeting strategies such as immunomodulatory monoclonal antibodies, adoptive cell transfers, high-dose immunostimulatory cytokines, and others (93). Among these, encouraging results have been achieved through the targeting of inhibitory crucial molecules of the antitumor T-cell response such as programmed-cell death 1 (PD-1) and cytotoxic T-lymphocyte-associated protein 4 (CTLA-4) (93).

As a downstream target of several receptors, Shp1 is also involved in T-cells signaling where it limits T-cell responsiveness either through direct dephosphorylation of the T-cell receptor (TCR)-ζ chain or dephosphorylation of downstream adaptor proteins including Lck, ZAP70, Vav, and PI3K (14, 94). Studies on CD8^+^ T-cells have demonstrated that the absence of Shp1 allows cells to form more stable and longer-lasting synapses with antigen presenting cells (APCs) (95), leading to reduced T-cells activation thresholds and to the stimulation and proliferation of low-affinity T-cells; this causes an increased number of tumor specific effector T-cells and a more efficient control of tumor growth (96, 97). Shp-1 depleted CD8^+^ T-cells result also to be more resistant to suppression by regulatory T-cells (T_reg_) (98), which is crucial for their survival into the tumor microenvironment. Similarly, also tumor-infiltrating lymphocytes (TILs) signaling has been reported to be regulated by Shp1, whose abrogation in TILs was found to restore TIL lytic function *in vitro* (99).

Shp1 has also been shown to inhibit T_reg_ suppressor function (100), and thus its inhibition in these specific cell population may support a tolerant microenvironment, promoting tumor growth and progression. Collectively, these data suggest the need to modulate Shp1 activity in specific and individual immune cell populations rather than perform a global inhibition of its activity, as obtained by systemic administration of Shp1 inhibitors. This consideration could also explain the disappointing results of the Phase I clinical studies discussed above.

Moreover, these findings raise the question of how modulation of TCR activation and signaling through Shp1 inhibition might improve responsiveness to existing checkpoint blockade immunotherapy. Several studies have indeed proven that Shp1 can be recruited to the cytoplasmic tail of PD-1 by binding to its immunoreceptor tyrosine-based switch motifs (ITSM) thus dephosphorylating and inactivating proximal signaling molecules of TCR activation (101, 102). Similarly, Snooke et al. have recently demonstrated that Shp1 knockdown potentiates the antitumor activity of T-cells responding to low-affinity tumor antigen, particularly in combinatorial studies blocking both PD-1 and CTLA-4 (97). Shp1 has also been successfully targeted in preclinical models of adoptive T-cell immunotherapy (103, 104). Here the abrogation of Shp1 in tumor-specific effector T-cells, either by *in-vitro* transduction of Shp-1 shRNA-expressing-T-cells (104) or T-cells conjugated with Shp1 inhibitor-loaded nanoparticles (103), significantly enhances the effector function and tumor clearance in the therapy of disseminated leukemia and advanced prostate cancer, respectively.

Therefore, these studies suggest that the inhibition of Shp1 in effector T-cells could be a therapeutic strategy to be used alone or in combination with other immunomodulating strategies (e.g., PD-1 or CTLA-4 inhibition) and amenable to increase therapeutic activity of the tumor-specific T-cells.

## Concluding Remarks

The above lines of evidence delineate Shp1 as a tumor suppressor gene contributing to tumorigenesis via the control of different cancer-related signaling and point at the potential of Shp1 as a target for therapy in a wide range of cancers. Synthetic lethality-based and rational combinatorial strategies are potential approaches to address the loss-of-function of Shp1 that occurs in several tumors and to restore the onco-suppressive activity of Shp1. Given the importance of genomic mechanisms regulating Shp1, epigenetic therapy through pharmacological modulation of histone modifications or promoter methylation could be particularly attractive. A few cancers are associated with elevated Shp1 expression; in this context Shp1 inhibition might represent an additional way to treat these tumors. Similarly, the role of Shp1 in tumor immunomodulation also inspires studies aimed at the identification and/or development of specific and safe Shp1 inhibitors.

The diverse Shp1 activities in different tumors demand for a detailed characterization of the Shp1-regulated pathway(s) altered in each specific case; similarly, the functional role of each Shp1 mutation needs to be functionally characterized in order to develop precise therapeutic approaches that will restore the Shp1 physiological action and hopefully provide improved cancer therapy.

## Author Contributions

AV, DS, and DC analyzed and discussed the data reported and wrote the manuscript.

## Conflict of Interest

The authors declare that the research was conducted in the absence of any commercial or financial relationships that could be construed as a potential conflict of interest.
